# Synthesis and evaluation of chitosan based controlled release nanoparticles for the delivery of ticagrelor

**DOI:** 10.1080/15685551.2022.2054117

**Published:** 2022-03-20

**Authors:** Nariman Shahid, Alia Erum, Muhammad Zaman, Ume Ruqia Tulain, Qurat-ul-ain Shoaib, Nadia Shamshad Malik, Rizwana Kausar, Ayesha Rashid, Umaira Rehman

**Affiliations:** a Faculty of Pharmacy, University of Sargodha, Sargodha, Pakistan; b Akhtar Saeed College of Pharmaceutical Sciences, Lahore, Pakistan; c Faculty of Pharmacy, University of Central Punjab, Lahore, Pakistan; d Faculty of Pharmacy, Capital University of Science and Technology, Islamabad, Pakistan; e ILM College of Pharmaceutical Sciences, Sargodha, Pakistan; f Department of Pharmacy, The Women University Multan, Pakistan

**Keywords:** Nanoparticles, chitosan, ticagrelor, hydrophobic, hydrophilic, ionic gelation, bioavailability

## Abstract

The aim of this contemporary work was to formulate a controlled release mucoadhesive nanoparticle formulation for enhancing the oral bioavailability of Ticagrelor (TG), a BCS class IV drug, having low oral bioavailability of about 36%. The nanoparticles can act as efficient carriers for hydrophobic drugs, due to having high surface area and hence can improve their aqueous solubility due to their hydrophilic nature. The nanoparticles (NPs) of TG were formulated using chitosan (CH) as polymer and sodium tripolyphosphate (TPP) as cross-linker, by ionic gelation technique with varying concentrations of polymer with respect to TG and TPP. Characterization of prepared nanoparticles was carried out to assess zeta potential, size, shape, entrapment efficiency (EE) and loading capacity (LC), using zeta sizer, surface morphology and chemical compatibility analysis. Drug release was observed using UV-Spectrophotometer. By increasing concentration of CH the desired size of particles (106.9 nm), zeta potential (22.6 mv) and poly dispersity index (0.364) was achieved. In vitro profiles showed a controlled and prolonged release of TG in both lower pH-1.2 and neutral pH-7.4 mediums, with effective protection of entrapped TG in simulated gastric conditions. X-ray diffraction patterns (XRD) showed the crystalline nature of formed NPs. Hence, this effort showed that hydrophobic drugs can be effectively encapsulated in nanoparticulate systems to enhance their solubility and stability, ultimately improving their bioavailability and effectiveness with better patient compliance by reducing dosing frequencies as well.

## 1. Introduction

The drug that belongs to BCS class IV has low solubility as well as permeability, due to which it faces the problem of poor bioavailability. Bioavailability is a crucial parameter for acquiring desired drug concentration in systemic circulation for therapeutic effect to be shown [1]. In recent years, various novel drug delivery techniques have been developed for improving solubility-related issues of poorly soluble drugs like size reduction methods, solid dispersion technique, lipid-based drug delivery systems, microparticles and nanoparticle technology, micellar technology etc. [2]. By formulating nanoparticles composed of naturally occurring biodegradable polymers, various solubility-related issues can be resolved. Nanoparticles containing naturally occurring biodegradable polymers have been revealed as promising carriers for controlled delivery of various therapeutic agents through the oral route [3].

By decreasing the size, surface area can be increased that results in easy wetting and rapid dissolution of the drug thereby enhancing drug solubility and ultimately its bioavailability. Nanoparticles reside in the systemic circulation for a longer period and release the consolidated agents at a predefined dose. Therefore, they produce negligible fluctuations in plasma with minimum adverse events [4].

Chitosan (CH) is a cationic polysaccharide and a biodegradable polymer from chitin family that has been extensively used for formulating nanoparticles for controlled delivery of various therapeutic agents orally [5]. CH possesses various characteristics of pharmaceutical interest due to its non-toxicity, biodegradability, biocompatibility and efficient drug loading capability. It permits a controlled and sustained effect of therapeutic moieties due to its slow biodegradation that can help in reducing dosing frequency and improving patient compliance. An additional advantage of using CH in nanoparticle formulation is its mucoadhesive property that can help in enhancing bioavailability of the drug. It acts as a penetration promoter as it opens tight epithelial junctions by facilitating paracellular as well as transcellular movement of drugs. CH associates with mucus membrane having negative charge by forming a complex due to ionic and hydrophobic interactions [6].

TG, an acyclopentyl triazolo pyrimidine, is a modern class of anti-platelet agents having anti-coagulant effect. It has been graded as BCS Class IV drug having poor solubility and permeability. TG has been reported to possess a limited oral bioavailability, about 36%. It reversibly binds to the P2Y12 receptor as it is a non-competitive, direct-acting P2Y12 inhibitor. It is prescribed to lower the rate of myocardial infarction, stroke and cardiovascular death in patients having acute coronary syndrome or myocardial infarction [7]. The usual dose administered is 90 mg b.d. The physicochemical properties of TG make it a suitable candidate to be formulated as nanoparticle [8]. Another advantage of formulating nanoparticles of TG would be the sustained and prolonged anticoagulant effect of TG [4].

## 2. Materials and methods

### 2.1 Materials

Chitosan having average molecular weight (100,000 g/mol) along with deacetylation degree of 75–85% and viscosity about 20–300cP (Sigma-Aldrich, USA), Glacial Acetic acid (Merck, Germany), HCl (Supelco, USA), NaCl (Supelco), Sodium tripolyphosphate (Sigma-Aldrich, USA), methanol (Supelco, USA), ethanol (Supelco, USA), analytical grade Acetonitrile (Sigma-Aldrich, USA), Ammonium acetate (Sigma-Aldrich, USA) and Ammonium hydroxide (Sigma-Aldrich, USA). TG was provided as a gift from CCL Pharmaceuticals, Lahore.

### 2.2 Preparation of TG loaded nanoparticles (TG-NPs)

**Figure 1.**
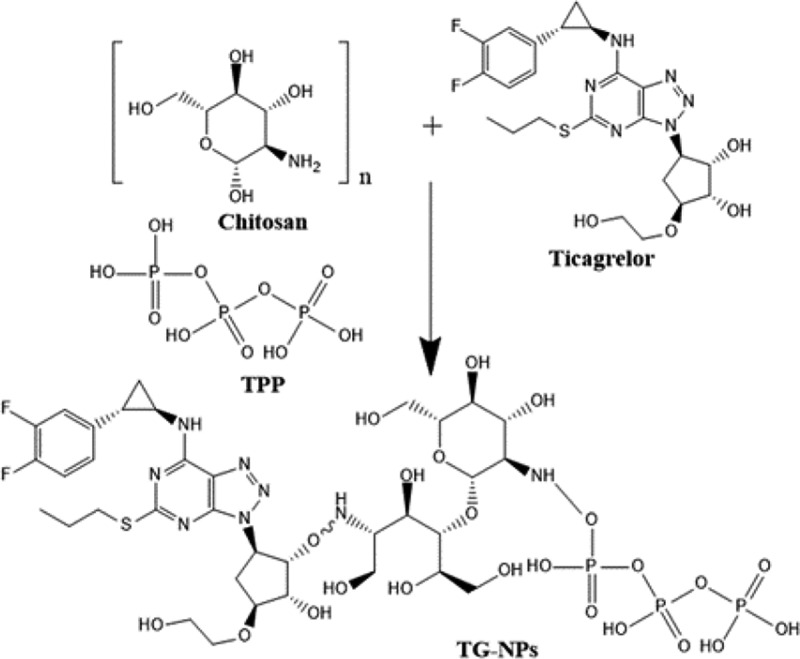
Possible grafted structure of TG loaded nanoparticles (TG-NPs).

CH nanoparticles loaded with TG were fabricated by ionic gelation technique by using Sodium tri-polyphosphate, TPP as a cross-linker, as reported by Tan Vui Nguyan and Reynaldo Esquivel [9]. CH was dissolved in 2%v/v acetic acid solution at varying concentrations, i.e., 1 mg/mL to 4 mg/mL. TG solution having 5 mg/mL concentration was added dropwise in CH solution (adjust pH to 4.5) followed by addition of 0.3% TPP solution dropwise under constant stirring at room temperature for 1 hour (Table 1). An opalescent suspension was then formed which was ultra-centrifuged at 12,000 RPM for half an hour. The settled nanoparticles were lyophilized at −50°C and were stored at 4°C for further use [10]. Figure 1 indicates possible grafted structure of TG-loaded nanoparticles.

**Table 1.** Composition used for the synthesis of TG-NPs

Formulations	TG: CH	CH: TPP
F1	1:1	1:1
F2	1:2	2:1
F3	1:3	3:1
F4	1:4	4:1

### 2.3 Physical characterization of TG-NPs

#### 2.3.1 Zeta size and Z-potential determination

Size of particles along with poly dispersity index (PDI) of TG-NPs was determined using Malvern Zeta sizer Nano ZS (Malvern, UK) having digital auto-correlator with a computation range (0.3 nm to 10 um), provided by temperature control unit, based on photon correlation spectroscopic (PCS) technique. The analysis was performed after further diluting NPs suspension, in triplicate.

Zeta potential was determined with Zetasizer Nano *Z* equipment having He-Ne laser light source at 633 nm, max 4 mW at measuring position of 2 mm at 25°C applying refractive index of dispersant (1.33), viscosity (0.8872 cP) and dielectric constant of dispersant (78.5). The samples comprising of a little amount of NP suspension were analyzed through clear glass cuvettes [11].

#### 2.3.2 Morphological characterization of TG-NPs

Structural morphology of TG-NPs with TG:CH weight ratio of 1:3 and 1:4 was carried out by Scanning Electron Microscopy (SEM) instrument (JSM-7610 F, JEOL). SEM is a constructive characterization technique that gives information regarding the surface features of the sample under observation, i.e., topography (surface characteristics of the particles) and morphology (particles’ shape, size and arrangement). SEM is comprised of a thermionic gun as an electron source along with other components [11].

#### 2.3.3 Evaluation of entrapment efficiency (EE) and loading capacity (LC) of TG-NPs

EE and LC of TG in NPs were assessed by quantifying the amount of unentrapped TG in supernatant solution after centrifuging the dispersion. TG nanoparticles’ entrapment efficiency (EE) was evaluated by ultrafiltration method [12]. Briefly, TG-NPs dispersion was ultra-centrifuged for 20 min at 12,000 rpm at 4°C, before freeze-drying. The supernatant solution was taken and analyzed by spectrophotometric analysis. The percentage obtained was taken as the free drug concentration. EE and LC of above-mentioned formulations were found by using following formulas:

$$EE\left(\%\right)=\left(\begin{array}{c} Total\;drug\;amount-\\ Free\;drug\;in\frac{NPs}{Total}drug\;amount \end{array}\right)\times 100$$

$$LC\left(\%\right)=\left(Total\;Drug\;amount-Free\;drug/NPs\;weight\right)\times 100$$

#### 2.3.4 Chemical compatibilities by Fourier transform infrared (FTIR) spectroscopy

FTIR of CH, TG and TG-NPs was performed using Digital FT-IR spectrometer (Bruker, Germany) equipped with diamond Attenuated Total Reflectance covering span of 8300–350 cm^−1^ along with interferometer having varying speed (0.1 to 4 cm/s) having IR diameter (2–11 mm). The IR spectra were taken (with a resolution of 4 cm^−1^) and observed to find out the molecular information about chemical structure and presence of various bonds [13].

#### 2.3.5 X-ray diffraction (XRD)

Crystallinity of TG as well as TG-NPs was assessed through PXRD pattern using Ni-filtered Cu-Kα radiation. Samples were scanned at an angle of 2θ starting from 5° to 120° at room temperature, and XRD patterns were collected. A step size of 0.02°/s was set for continuous scanning [14].

#### 2.3.6 DSC analysis

For evaluating the energy changes in pure TG, TG-NPs and physical mixture, thermal analysis was conducted by differential scanning calorimetry (DSC), using a thermal analyzer (DSC Q2000 V24, Seoul, Korea). DSC is a thermoanalytical procedure in which the difference in the amount of heat needed to increase the temperature of a sample and reference material as a function of temperature is evaluated. A small quantity of sample (5–10 mg) was added in separate aluminium pans that were heated from 30 to 310 °C. DSC thermograms were recorded at a heating rate of 20°C/min under nitrogen flow (20 mL/min) [15].

#### 2.3.7 Evaluation of invitro release of TGNPs

An in vitro release study was executed by dialysis method. TG-NPs were dissolved in 5mL dissolution medium (pH 1.2 buffer and phosphate-buffered saline of pH 7.4 having 0.1%w/v Tween 80) separately, in dialyzing membrane (Mw cutoff 12kDa). The sealed dialysis bag was submerged in 100-mL release medium under constant stirring at 150 rpm, maintaining the temperature at 37°C. For increasing the solubility of TG in the buffer medium, Tween 80 was added. After appropriate intermissions, samples were taken out and restored by the same quantity of release medium. The amount of TG released from the nanoparticles was evaluated by photometric analysis at 255 nm.

The calibration curves of TG at both pH 1.2 and pH 7.4 were drawn to find out unknown drug concentration [16].

#### 2.3.8 In vitro kinetic modelling of drug release

Various in vitro kinetic models such as first-order, zero-order, Higuchi, Korsmeyer-Peppas and Hixson Crowell were applied to the obtained data to assess drug release pattern and kinetics of TG-NPs. In vitro release study was performed in triplicate and kinetic modelling was performed on the mean values obtained from the release study at different time intervals [16].

## 3. Results and discussion

### 3.1 Effect of CH toTPP mass ratio on TG-NPs formation

#### 3.1.1 Effect on particle size

The impact of TPP, having different mass ratio with that of CH (1:1 to 4:1, CH:TPP), on the particle size was studied (Table 2). TPP and CH mass ratio significantly influences the characteristics of TG-NPs. The particle size decreased with the increasing CH-TPP mass ratio from 1:1 to 4:1. The observed size of F1 was maximum, i.e., 646.4 nm while that of F4 was minimum, i.e., 106.9 nm. Hence by increasing the concentration of CH in comparison with drug and TPP, the particle size of formed nanoparticles decreased accordingly. CH chains are comprised of large number of reactive amine groups that act as prospective crosslinking positions for negatively charged polyanions (Phosphate groups) of TPP. Therefore, molar concentration of TPP and CH is interlinked to degree of crosslinking, but that shows limitation due to phenomena of aggregation at large polyanion concentration. Hence, it can be observed that with the same molar ratio of TPP and CH, i.e., 1:1, particle size became large (646.4 nm) as compared to that having low concentration of TPP in comparison to CH, i.e., 4:1(106.9 nm). This characteristic is indicating that even at low concentration of polyanions of TPP as compared to cations of CH, enough ionic interactions have taken place which resulted in formation of small-sized nanoparticles due to ionic gelation. As CH-TPP mass ratio declined, the quantity of TPP available for ionic interactions increased and the surplus TPP would has linked the monoparticles to form larger nanoparticles.

**Table 2.** Effect of CH concentration on particle size, zeta potential and PDI

Formulations	Particle size (nm)	Zeta Potential (mv)	PDI	EE (%)	LC (%)
F1	646.4	−4.39	0.082	96.9	52.09
F2	328.6	−1.75	0.435	97.18	40.5
F3	175.2	23.2	0.518	85	42.5
F4	106.9	22.6	0.364	84.1	42.05

#### 3.1.2 Effect on zeta potential

The effects of different mass ratio of CH:TPP (1:1 to 4:1) upon NPs’ zeta potential were analyzed. As per results (Table 2), a prominent increase of zeta potential was observed by decreasing the amount of TPP. Zeta potential increased proportionally by increasing the CH concentration due to neutralization of charge between negatively charged TPP and positive amine groups of CH. Zeta potential indicates the repulsion or attraction among adjoining particles. Value of zeta potential increased from −4.39 for F1 to +22.6 for F4. As F1 contained more poly-anions, the zeta potential was observed to be negative, while as the concentration of poly-anions decreased and concentration of cations increased in F4, the zeta potential became positive that might be a consequence of less ionic interactions. As CH is a cationic polymer, it contains positive charges that can bring about powerful electrostatic interlinkage with mucus membrane possessing negative charge, therefore nanoparticles with a positive zeta potential would exhibit relatively more prominent mucoadhesion. This type of interaction is advantageous for strong adhesion as well as immediate immobilization on negatively charged membranes [10]. Moreover, particle size as well as zeta potential of formulations F3 and F4 were in the desired range. So, further characterization was done on these two formulations.

#### 3.1.3 Morphology

**Figure 2.**
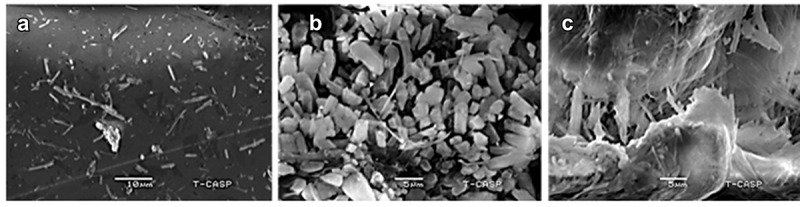
SEM images of TG NPs (a) TG loaded CH NPs at 1:3 (F3), (b) TG loaded CH NPs at 1:4 (F4), (c) Pure CH.

SEM images of TG-NPs were obtained. Figure 2 showed the SEM micrographs The images demonstrated that the TG-NPs having low concentration of CH in F3(2A) possessed a more elongated structure with average particle size of 175.2 nm as compared to NPs having high concentration of CH in F4(2B) that also demonstrated an oblonged morphology with an average particle size of 106.9 nm. At molar ratio of CH:TPP (3:1), an increase in size was observed probably due to excessive concentration of TPP that limited the intramolecular crosslinking when compared with that having low concentration of TPP versus CH (4:1). A change in morphology was observed by changing the TPP concentration and CH concentration. Figure 2C displayed the morphology of pure chitosan depicting a rough and irregular surface. Whereas addition of TPP to the TG and CH conjugate has resulted in the formation of discrete NPs with uniform morphology. Moreover, by increasing the concentration of CH, the NPs with improved uniform shape were obtained as depicted in Figure 2b as compared to that of Figure 2a. Hence, it can be concluded so as it is possible that size, charge and morphology of NPs can be modulated from 100 nm and 1000 nm by varying the concentration of CH with respect to TPP and TG.

### 3.2 Entrapment efficiency and loading capacity

Free drug concentration, which has been estimated by HPLC for F1, F2, F3 and F4, was found to be 3.12%, 14.1%, 15.3% and 15.9%, respectively (Table 1). EE of F1, F2, F3 and F4 was found to be 96.9%, 97.18%, 85% and 84.1%, respectively. The loading capacity of F1, F2, F3 and F4 was found to be 52.09%, 40.5%, 42.5% and 42.05%, respectively. Ideally, an efficient delivery technique must possess a greater degree of association with drugs. EE was found to be greater in those formulations having low concentration of CH in comparison with TPP and drug and vice versa. However, EE for all formulations was fairly good, indicating the fact that enough ionic interactions were present between positively charged CH and negatively charged TPP that resulted in efficient entrapment of drug within the formed nanoparticles. The high EE could be due to the hydrophobicity of TG, enabling it to enter the hydrophobic interior of the TG-NPs.

Similarly, drug loading capacity was found to be greater (52.09%) for the formulation having low concentration of CH, that decreased to some extent (≈42%) for nanoparticle formulations having its higher concentration. A better drug loading capacity was expected due to the hydrophobic nature of TG. Moreover, there are multiple ionic sites available on TG that can facilitate the incorporation of TG into the TG-NPs.

Loading capacity might has decreased due to less availability of anions for interaction as the concentration of TPP decreased subsequently in these formulations, F3 and F4, respectively.

### 3.4 Fourier transform–infrared (FT-IR) spectroscopy

**Figure 3.**
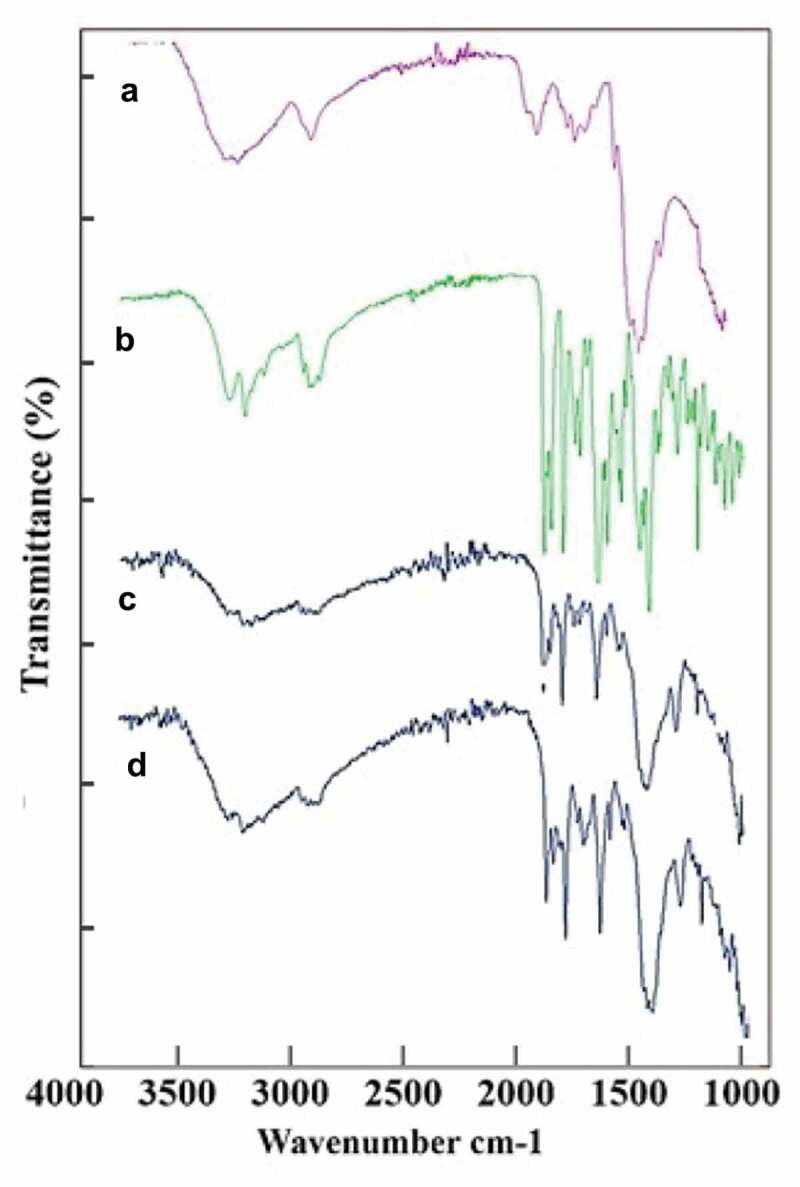
FTIR images of (a) Pure CH (b) TG (c) F3 and (d) F4, where all the corresponding peaks are visible in the IR scan, indicating chemical compatibilities of selected ingredients.

FT-IR spectrums of pure CH, pure drug (TG) as well as the two formulations, i.e., F3 & F4 were taken to find out the chemical compatibilities and bonds formed due to formation of nanoparticles (Figure 3). FTIR spectrum of pure CH displayed major peaks at 3405, 3340,2869,1621, 1584, 1401, 1378, 1029, 1013 and 896 cm^−1^. The absorption bands at 3405 and 3340 cm^−1^ are indicative of the presence of hydroxyl groups (-OH) while the peak at 2869 cm^−1^ is showing the alkyl/methyl stretch (-C-H). The intense peaks at 1621 and 1584 cm^−1^ are indicating presence of amine groups (-NH_2_). Additional absorption bands at 1013 and 896 cm^−1^ are characterizing polysaccharide structure of CH.

The FTIR spectrum of pure TG showed major absorption bands at 3405, 3286, 2856, 1601, 1560, 1506, 1451, 1404, 1312, 1250, 1209, 1112, 1051 and 755 cm^−1^. The absorption bands at 3405 and 3286 cm^−1^ indicated the existence of -N-H stretch and -O-H stretch. Two to three peaks with medium intensity in the range 2800 to 2900 cm^−1^ specified the alkyl (–C-H) stretch existence. The absorption bands at 1561 and 1600 cm^−1^ expressed the appearance of – N-H stretch. The peaks at 1404 and 1451 cm^−1^ are showing methyl bend, at 1209 and 1250 cm^−1^ are showing – C-OH stretch, whereas at 1051 and 1112 cm^−1^ are reflecting presence of – C-O stretch. Moreover the major peaks observed in formulations F3 and F4 were at 3314, 2960, 1716, 1624, 1540, 1327, 1245, 1068, 1039 and 880 cm^−1^. The observed intense band at 3314 cm^−1^ corresponded to – O-H stretch, whereas strong intense peak at 2960 cm^−1^ reflected the presence of methylene (-C-H stretch). The peak at 1624 cm^−1^ corresponding to C-NH stretch was also observed in F3 and F4 as observed in pure CH. The intense peak at 1327 cm^−1^ corresponded existence of – C-F stretch. Several peaks observed between 1050 and 1250 cm^−1^ confirmed the – C-O stretch. A peak at 880 cm^−1^ represented bisulphide linkage (–S-S).

### 3.5 X- Ray diffraction (XRD)

**Figure 4.**
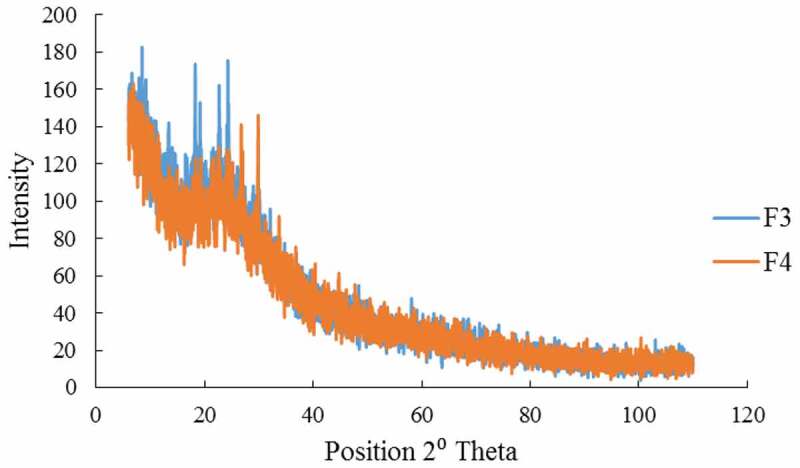
XRD spectra of TG loaded CH-NPs (F3 & F4).

The characteristic diffraction peaks showed by the formulations F3 and F4 indicated crystalline nature of the nanoparticle formulations. Intense diffraction peaks have been observed in F3 and F4. The diffraction peak of pure CH usually observed at 20.2° has slightly shifted to 19.19° and 19.24° in F3 and F4, respectively, which may be indicative of ionic interaction of CH with TPP and crystalline nature of the nanoparticles formed. The peak intensity of CH decreased in the nanoparticle formulations F3 and F4 (Figure 4). The intensity of diffraction peak of F4 is low at 19.24° as compared to F3, which may indicate the amorphous nature of the nanoparticles. The absence of any other diffraction peak corresponding to impurities reflects the purity of the formulations.

### 3.6 Diffraction scanning calorimeter (DSC)

**Figure 5.**
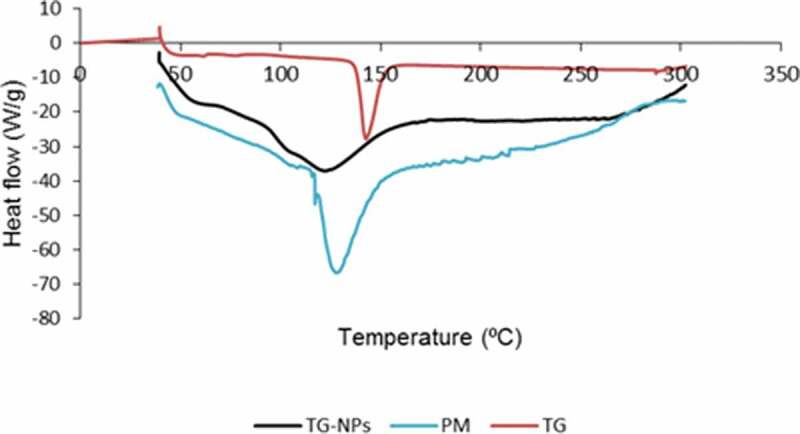
DSC thermograms of pure Ticagrelor (TG), Physical mixture (PM) and TG-NPs.

DSC thermograms for pure TG, physical mixture (PM) and TG-NPs were recorded (Figure 5). Pure TG displayed an endothermic peak at 142.74 °C indicating the melting point of TG. The physical mixture showed the original peaks indicating no interaction among components. TG-loaded chitosan-based NPs presented a broader peak along with the reduction in melting point as shown by an endothermic peak at 122.13 °C. This slight reduction in melting point may be attributed to the interaction between TG and CH that has brought about the broadening of TG’s melting peak with a shift at lower temperature. This can also be attributed to the reduction in the degree of crystallinity of encapsulated TG due to the formation of nanoparticles.

### 3.7 In vitro release studies

A slow release behavior of TG-NPs at both pH 1.2 and pH 7.4 was observed as compared to that with pure drug, which has shown a 100% release in less time as previously reported [15].

**Figure 6.**
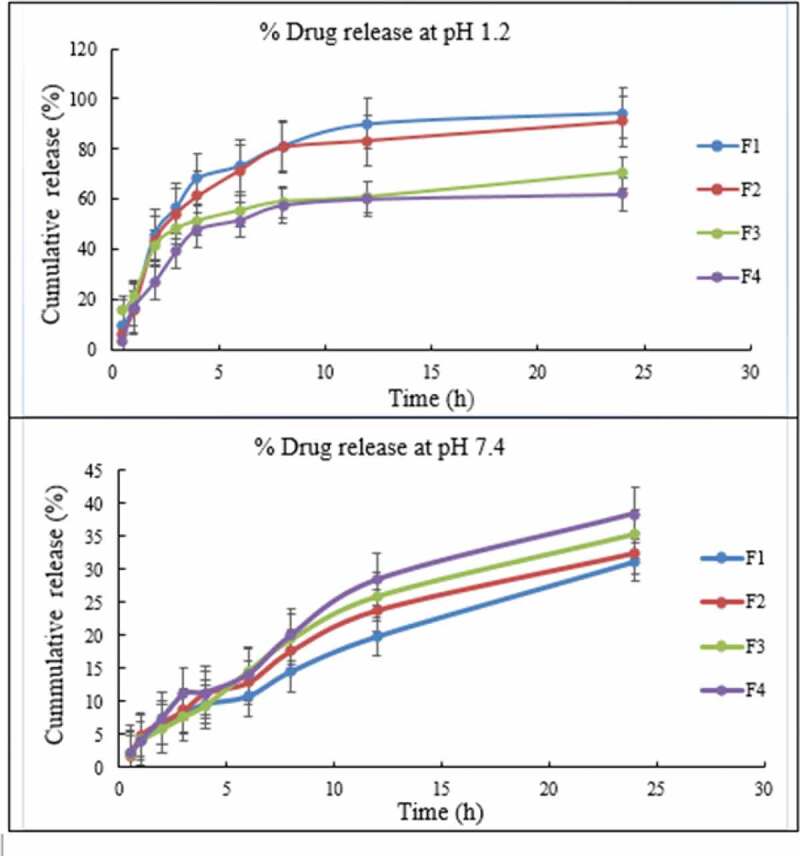
*In-vitro* drug release profile of TG loaded CH NPs at pH 1.2 and pH 7.4, illustrating sustained and controlled release of drug.

At pH 1.2 the formulations F1, F2, F3 and F4 showed a maximum release of 94.4%, 90.88%, 70.58% and 61.8%, respectively, after 24 hours. F3 showed a burst release of about 16% in first 30 minutes followed by a prolonged release of about 69.8% in 24 hours (Figure 6). F4 showed a slow release pattern of about 27% in first 2 hours followed by a prolonged release of up to 61.8% in 24 hours. TG was released slowly in the later stage because of slow polymer degradation or swelling. Remaining part of TG in CH NPs was not entirely released that might be due to the fact that the particles were not fully disintegrated or dissolved in the dissolution medium. This might has happened due to the interaction among the free amino groups on the CH fragments and residual TG [17]. This characteristic may be beneficial in a sense that it will prevent the drug from degradation in acidic environment of stomach and would enhance intestinal absorption of TG in the form of nanoparticles as more of the undissolved drug will move towards the small intestine.

While at pH 7.4, the formulations F1, F2, F3 and F4 showed a maximum release of 31.11%, 32.47%, 33.29% and 38.42%, respectively, after 24 hours. The slow and less release of TG at neutral pH might be due to the fact that TG is a basic drug, which dissolves well at acidic pH as compared to that at neutral or basic pH [12]. As TG can be advised to a patient for prolonged period of up to 12 months as a preventive therapy, therefore, sustained effect for prolonged period of time will be beneficial in this regard. In a multiple-dose study conducted earlier in healthy volunteers, it was observed that inhibition of platelet aggregation (IPA) slowly decreased as plasma concentration declined after 12 hours of dosing, indicating that IPA associated with TG is concentration-dependent and is reversible as well [18]. Here, a slow and sustained release of TG in once daily dosing will prevent reversal of effect and will be beneficial in prolonging its antiplatelet activity and patient compliance by reducing the dosing frequency.

This result was supported by the previous study in which the cumulative release of prepared chitosan NPs over 24 hours study period was found to be in the range of 51.57% to 69.93% depicting a sustained release behavior [19].

### 3.8 Evaluation of drug release kinetics

In vitro release kinetics were assessed to ascertain best suitable release behavior of TG loaded nanoparticle formulations (F1-F4). The in vitro release data achieved was subjected to various kinetic models such as first-order, zero-order, Korsmeyer-peppas, Higuchi and Hixson-crowell. From the kinetic modelling data, it was revealed that TG loaded NPs followed both Higuchi and Korsmeyer-Peppas model due to high correlation coefficient values obtained in both cases. Upon comparison of R^2^ values, Korsmeyer-peppas model was predicted as the best fit model. Obtained results displayed that the release of TG from CH-based NPs was diffusion controlled proving as a tool toward achieving sustained release of drug from the polymeric NPs. An anomalous mechanism for drug release was followed by NP formulations based upon diffusion and swelling of polymer as well. As n (diffusion coefficient) value was observed to be greater than 0.45, it can be assured that the drug released from the NPs by non-fickian diffusion mechanism. The results obtained by applying various kinetic models have been represented in Table 3, where k values represent the kinetic constants.

**Table 3.** Correlation coefficients (R^2^) and release rate constants of TG loaded nanoparticle formulations (F1-F4)

		F1	F2	F3	F4
Zero-order	R^2^	0.9903	0.9691	0.9686	0.9707
	k0(%/h)	1.465	1.614	1.74	1.89
First-order	R^2^	0.9956	0.9829	0.9842	0.9858
	k1(h^−1^)	0.018	0.02	0.022	0.024
Higuchi	R^2^	0.9914	0.9951	0.9919	0.992
	kH(%/h^1/2^)	5.562	6.211	6.61	7.208
Korsmeyer- peppas	R^2^	0.9976	0.9939	0.9906	0.9916
	kKP(h^−n^)	3.681	4.603	4.339	4.939
	N	0.67	0.624	0.674	0.656
Hixson-crowell	R^2^	0.9943	0.9789	0.9798	0.9817
	kHC(%^1/3^/h)	0.006	0.006	0.007	0.007

## 4. Conclusion

The attempt to fabricate the sustained release nanoparticles of TG was found successful, as nanosized particles (20–200 nm) were observed through, both zeta size analysis and SEM photographs. The sustained release was being observed for 24 hrs, confirming the once daily dosing of TG to enhance the patient compliance. Moreover, as future prospect, the kinetic study for TG release can be performed in the presence of enzymes as well to observe the drug release behavior in a better way. In conclusion, in the current study, fabricated nanoparticles loaded with TG displayed encouraging results for developing an effective drug delivery mode for oral administration of antiplatelet agents.
